# LGBT communities and substance use in Queensland, Australia: Perceptions of young people and community stakeholders

**DOI:** 10.1371/journal.pone.0204730

**Published:** 2018-09-27

**Authors:** Daniel Demant, Leanne Hides, Katherine M. White, David J. Kavanagh

**Affiliations:** 1 School of Psychology and Counselling, and Institute of Health and Biomedical Innovation, Faculty of Health, Queensland University of Technology, Brisbane, Queensland, Australia; 2 Australian Centre for Public and Population Health Research, Faculty of Health, University of Technology Sydney, Ultimo, New South Wales, Australia; 3 School of Psychology, Faculty of Humanities and Social Sciences, The University of Queensland, Brisbane, Queensland, Australia; The Hong Kong Polytechnic University, HONG KONG

## Abstract

Sexual minority young people use licit and illicit substances at disproportionate levels. However, little is known about the perceptions of substance use among members of LGBT communities. This paper reports the results of a content analysis of 45 semi-structured interviews about substance use in LGBT communities with sexual minority young people (n = 31) and community stakeholders (n = 14). Results indicated both sexual minority youth and community stakeholders perceived the use and acceptance of substances to be higher in LGBT communities compared to the general population. Participants identified a range of characteristics potentially leading to higher levels of substance use including peer pressure, high exposure to substance use, and the high concentrations of licensed venues in LGBT communities. Marginalisation, discrimination and mental health were also perceived as important reasons for these disparities. Community stakeholders identified a range of potential interventions including legislation to address discrimination and substance use, increased services and activities, advertising in commercial LGBT venues and social media, and reinvigorating community cohesion.

## Introduction

Sexual minority adolescents and young adults have worse health outcomes than their sexual majority peers [1] including disproportionate levels of alcohol [2], tobacco [3], and illicit substance use [4, 5]. Research has shown that certain substances such as methamphetamine or GHB (gamma hydroxybutyrate) are used at particularly high rates among gay and bisexual men for sexual sensation seeking [6, 7], particularly among those living with HIV or AIDS [8]. While the body of research on the health of sexual minorities is growing, the majority of current research focuses on sexual health among gay and bisexual men and other men who have sex with men [9].

Literature on the underlying reasons for the disparities in substance use between sexual minority adolescents and young adults, and their sexual majority counterparts is scarce [9]. This is alarming, given the significantly higher rates of harmful substance use among sexual minority youth compared to their heterosexual counterparts in studies conducted to date [10, 11].

There is general consensus that the increased risk of substance use among LGBT youth is likely to be the result of a combination of individual, community, and societal factors.

Models such as the minority stress model link the increased risk of substance use to the discrimination, marginalisation and oppression sexual minority groups endure in often homonegative and heterosexist environments [12]. In this context, substance use is generally considered to be a coping mechanism for proximal and distal stressors associated with sexual minority statuses [13–16]. Research also indicates that sexual minority youth are more sensitive to the negative effects of discrimination and oppression at an age that coincides with the typical age of onset of substance use [4, 11, 15].

LGBT communities were also been identified as potential contributors to the disproportionate levels of substance use in sexual minority populations. While research is limited, results suggest that LGBT communities play an important role in the life of sexual minority people, providing a safe environment that offers protections against a society perceived as heterodominant and homonegative [17–19]. Lelutiu-Weinberger et al. [20] found evidence that identification and connectedness with LGBT communities may be protective against problematic substance use among young gay and bisexual men but appears not to be protective among older gay and bisexual men. Other studies showed that very low or very high levels of identification and participation with LGBT communities may be associated with elevated substance use among gay and bisexual men whereas men with moderate affiliations show considerably lower rates of substance use [21, 22]. However, no published research on the role of LGBT communities on substance use among sexual minority women could be identified, highlighting the need for further research.

One potential moderator of substance use, particularly of alcohol use, may be the physical manifestation of LGBT communities, which often revolve around parties and licensed venues such as bars, clubs, discos, and pubs [23]. Sexual minority people spend more time in licensed venues than their heterosexual counterparts as these venues are safe spaces to socialise with sexual minority peers [23]. The constant availability of alcohol and the higher levels of acceptability and illicit drug use in this nightlife economy are likely to increase substance use [24–28]. This factor also increases exposure to the targeted marketing campaigns of alcohol and tobacco companies to sexual minority populations [29, 30]. However, the majority of research to date on the role of LGBT communities on substance use itself is limited to descriptive studies. A more in-depth understanding of cultural factors and attitudes towards substance use in sexual minority young people is required to increase current understanding of the influence of LGBT communities on substance use levels.

This paper aimed to explore perceptions of the extent (prevalence) and acceptability of substance use in sexual minority populations. A further objective was to explore reasons for both substance use and substance use disparities among sexual minority populations, including the role of LGBT communities, among sexual minority young people. Self-identified stakeholders within LGBT communities (with professional experience working for an LGBT NGO or for-profit health or licensed venues) were then asked to comment on the young people’s perceptions about substance use within LGBT communities, and what types of interventions may help to reduce harmful levels of substance use in sexual minority young people. The inclusion of stakeholders in this study provides important insights from a group of experts with knowledge of both the culture of LGBT communities and substance use within it. It further allowed for a critical examination of young participants’ perceptions.

## Materials and methods

### Recruitment and participants

Two different groups of participants were recruited in Queensland, Australia, for this research: sexual minority young people (group I) and self-identified stakeholders within LGBT communities (group II). All participants were required to provide informed consent and have an adequate understanding of written and verbal English. Additionally, participants in group I were eligible to participate if they (i) were aged 14 to 35 years, (ii) had a sexual minority identity, and (iii) had used at least one licit or illicit substance in their lifetime (see Table 1). Participants in group II were eligible if they (i) were over the age of 18 years and (ii) self-identified as stakeholders within LGBT communities. Stakeholders were required to possess professional experience working for an LGBT non-government organisation (NGO) or an LGBT for-profit, licensed, venue. An LGBT for-profit venue was defined as a venue that is either widely recognised as primarily serving LGBT communities or identified itself as part of an LGBT community. A convenience sampling approach was used; participants were recruited via social media, email lists, and community-based organisations working with LGBT communities. Snowball sampling techniques were also used. Recruitment texts stated that the study is part of a wider research program on substance use in sexual minority young people.

### Procedures

Separate interviews were conducted for the two groups. Interviews with sexual minority young people (group I) were conducted between May 2015 and January 2016. Interviews with stakeholders were conducted between April and July 2016. Study procedures were designed to enable the use of a content analysis approach for both groups. Most interviews were conducted face-to-face in a private university office with five stakeholders interviews conducted via telephone. All interviews were audio recorded and transcribed strictly verbatim for a closer representation of the true interview situation and to ease access to latent content [31], field notes were made during interviews to check for consistency. Ethical approval to conduct the study was obtained from the Queensland University of Technology Human Research Ethics committee (Approval number: 1500000095).

Group I. A brief online survey was completed prior to the interview to check eligibility and collect information about demographics and substance use. A semi-structured interview was developed using the current literature on substance use in sexual minority populations and LGBT communities. Participants were asked open-ended questions about their participation in and view of LGBT communities (e.g., “How would you describe your links to the LGBT community?”) as well as questions about the use and acceptability of substance use in these communities (e.g., “How would you describe substance use in the LGBT community?”) and potential reasons for use (e.g., “Why do you think LGB/sexual minority people take substances?”). The terms ‘substance use’ and ‘LGBT community’ were not predefined for participants. An LGBT-specific ethical strategies including the use of an out gay interviewer were applied to ensure participants were provided with an empathic, accepting and safe environment to speak openly about their experiences with and perceptions of substance use in LGBT communities [32].

Group II. A semi-structured interview was developed using themes gathered from the content analysis of interviews with group I participants. Participants were also asked open-ended questions about their perceptions of the acceptability and reasons for substances use in LGBT communities. In the second part of the interview, participants were provided with short summaries of the thoughts and ideas of interviews from group I and asked about their opinions and professional experiences with substance use in the communities, particularly in sexual minority young people.

### Analysis approach

For both groups, a conventional content analysis [31, 33] was conducted to identify main categories and subcategories with definitions and coding rules for each category [34]. Content analysis approaches are high in reliability and validity even though there is no strict set of steps for content analysis, and different approaches are regularly undertaken and accepted in (public) health research [34, 35]. The analysis followed Pope et al.’s [36] 5-step approach: (i) broad familiarisation with the data (listening to interview recordings, reading transcripts), (ii)identifying a thematic framework (identifying key themes emerging from the data), (iii) indexing the data based on the thematic framework, (iv) charting and rearranging the data, and in a last step (v) mapping and interpreting the data.

Semi-structured interview questions were used to strengthen the validity of a content analysis approach [31]. The analysis focussed on latent and manifest content to ensure that the preconceived research questions as well as emergent themes were covered, using the individual participant as the unit of analysis [37]. Thematic saturation was reached after 31 and 14 interviews in group I and II, respectively. The analytic software NVivo10 was used to code the data line-by-line to strengthen the reliability of the analysis [38]. Interviews were re-coded for potential latent content if a new category or subcategory emerged at a later point. Several steps have been taken to strengthen the quality of analysis. Responses were validated by summarising important information collected via field notes to participants throughout the interviews and asking if the interviewer’s perceptions were correct or misinterpreted. Information about substance use provided by all participants in group I in a survey prior to the interview was cross-checked with transcribed interviews to check for data consistency. Any identifiers were deleted immediately after the interview and transcripts were therefore not returned to participants for comments.

The first author re-coded five randomly selected interviews a second time to test for potential coding differences, but no meaningful differences were detected. The co-authors read and commented during and after the initial analysis, reaching consensus about the final analysis. Finally, an independent researcher coded the main categories from 5 randomly selected interviews of group I and 4 randomly selected interviews from group II [39]. Only negligible differences in coding were identified, and were fully resolved in face-to-face meetings. A consolidated criteria for reporting qualitative studies (COREQ) 32-item checklist [40] is available as supplementary material (see S1 File).

### Interview and participant characteristics

Group I. The median interview length was 31 minutes (range: 17 to 55 minutes). Table 1 outlines the characteristics of all participants. Participants ranged in age from 18 to 34 years with a mean age of 24.4 (*SD =* 5.1) years. Fifteen participants identified as female, 13 as male, two as non-binary and one as genderqueer. The majority of participants identified as lesbian or gay (*n* = 19) followed by bisexual (*n* = 9), queer (*n* = 2), and pansexual (*n* = 1). All but four participants identified their ethnicity as White or Caucasian. The sample had a high level of education, including 13 young people with a university degree and 15 current tertiary students. Nearly all participants (*n* = 30) had consumed alcohol in the past 12 months and eight had consumed tobacco products. Twenty-one participants had used illicit substances at least once in their lifetime and 17 of those had used an illicit substance in the past 12 months.

**Table 1:** Group I participant characteristics.

	Age	Gender identity	Sexual identity	Ethnicity	Substance Use experiences			
					Alcohol*	Tobacco*	Illicit*	Illicit^#^
**1**	25	Male	Gay	Indian	X	X	X	X
**2**	20	Female	Lesbian	White/Caucasian	X	-	-	-
**3**	24	Female	Lesbian	White/Caucasian	X	-	-	X
**4**	20	Non-binary	Bisexual	White/Caucasian	-	-	X	X
**5**	20	Female	Bisexual	White/Caucasian	X	-	X	X
**6**	30	Female	Bisexual	White/Caucasian	X	X	X	X
**7**	18	Male	Gay	White/Caucasian	X	-	X	X
**8**	29	Male	Gay	White/Caucasian	X	-	X	X
**9**	28	Male	Gay	White/Caucasian	X	-	X	X
**10**	19	Male	Gay	White/Caucasian	X	X	X	X
**11**	28	Male	Gay	White/Caucasian	X	-	-	-
**12**	31	Female	Queer	White/Caucasian	X	X	X	X
**13**	22	Female	Lesbian	White/Caucasian	X	-	-	X
**14**	25	Female	Bisexual	Asian	X	-	-	-
**15**	21	Male	Gay	White/Caucasian	X	X	X	X
**16**	22	Male	Gay	White/Caucasian	X	-	X	X
**17**	33	Male	Gay	White/Caucasian	X	X	X	X
**18**	31	Female	Bisexual	White/Caucasian	X	-	-	X
**19**	24	Female	Bisexual	White/Caucasian	X	-	-	-
**20**	24	Male	Gay	White/Caucasian	X	-	-	-
**21**	19	Female	Lesbian	White/Caucasian	X	-	-	-
**22**	34	Female	Lesbian	White/Caucasian	X	-	-	-
**23**	19	Non-binary	Gay/Lesbian	White/Caucasian	X	X	X	X
**24**	18	Male	Pansexual	White/Caucasian	X	-	-	X
**25**	34	Male	Gay	Asian	X	-	-	X
**26**	20	Female	Bisexual	Asian	X	-	-	-
**27**	28	Genderqueer	Bisexual	White/Caucasian	X	-	-	-
**28**	21	Male	Gay	White/Caucasian	X	-	X	X
**29**	19	Female	Lesbian	White/Caucasian	X	X	X	X
**30**	21	Female	Queer	White/Caucasian	X	X	X	X
**31**	29	Female	Bisexual	White/Caucasian	X	-	-	-

* past 12 months.

^#^ lifetime use.

X indicates use.

Group II. The median interview length was 31 minutes (range: 15 to 37 minutes). Participants ranged in age from 31 to 57 years with a mean age of 46.7(*SD = 8*.*7*) years. Only two participants identified as female, and all other participants identified as male. Eight participants had professional experiences working for LGBT (health) organisations, four participants for LGBT for-profit venues, and two possessed professional experience working for both LGBT (health) organisations and LGBT for-profit venues. Length of professional experience ranged from 3 to over 20 years, with 8 participants having more than 10 years’ experience and 5 participants having at least 5 years. Further detailed information cannot be provided to ensure the anonymity of stakeholders.

## Results

### Perception of the extent and acceptability of substance use in LGBT communities

Table 2 provides a brief overview of all themes and sub-themes that emerged from the data. In the first part of the interviews, all sexual minority young people were asked about their perceptions of the extent and the acceptability of substance use within LGBT communities. Participants reported a wide range of involvement in LGBT communities, with some participants rarely participating and others participating regularly to very often.

**Table 2:** Overview of themes.

**Main theme 1:** **Perceptions of the extent and acceptability of substance use in LGBT communities**	
A significant problem	
Higher visibility and prevalence	
Stereotyping and media bias	
Specific substances	
Higher degree of acceptability	
**Main theme 2:** **Reasons for disparities in Substance use**	
LGBT culture and life	Role of substance use in socialising
	Peer pressure
Socio-political and ethical values of LGBT communities	Cultural norms and religion
	Liberty
Marginalisation, discrimination, and mental health	Being different
	Isolation and belonging
	Discrimination
	Mental health
Other reasons	Media influence
	Inherited behaviour
	Demographic differences
	Conflicting identities
**Main theme 3:** **Potential interventions**	
Politics and legislation	
LGBT NGOs and existing activities and groups	
Commercial LGBT venues	
LGBT communities	

Different subcategories emerged from the data: higher visibility and prevalence, stereotyping and media bias, the use of specific substances, higher degree of acceptability, and the acceptability of specific substances.

#### A significant problem

The majority of sexual minority young people considered substance use to be a major problem within LGBT communities and perceived LGBT young people to have higher rates of substance use and to be more experienced in using a range of licit and illicit substances than the general population.

*“I think it’s widespread […] I would like it not to be synonymous with the gay community or to be more prevalent*. *It would be good to see a downturn*.*”* (Sexual Minority Young Person 25)

*“I think all my gay friends have done drugs*. *I can’t say all of my straight friends have*.*”* (Sexual Minority Young Person 16)

While substance use was discussed in general, participants made no clear distinction between harmful and non-harmful levels of substance use. The perceived extent of the problem is highlighted by some sexual minority young peoples’ perceptions of the need to estrange themselves from LGBT communities to avoid substance use.

*“I think I possibly engage less now [in the LGBT community] because of the links to illicit drugs because I feel like I need to separate myself from those friends because I have things to do that don’t work well with that lifestyle choice*.*”* (Sexual Minority Young Person 6)

This perception was echoed by stakeholders who overall considered licit and illicit substance use to be a significant problem within LGBT communities including higher levels of high risk and harmful use.

*“They are used prevalently and I believe that we have a problem in our community*.*”* (Stakeholder 7)

#### Higher visibility and prevalence

Even though sexual minority young people noted that substance use is also present in the general culture and in licensed venues not specifically catering to LGBT communities, it was perceived to be more prevalent and visible within LGBT venues including harmful and multi-drug use.

*“When I go out clubbing at a straight venue and not a community venue*, *it’s still pretty bad*, *but you don’t actively see people running around selling drugs*, *seeing them take it*, *bragging about it and just generally they seem a bit less intoxicated*. *People are more regular*. *Yeah*, *it’s not as boastful as it is in the gay community […] at the gay venue it’s like ‘Yeah*. *I’ve been drinking and I’ve smoked weed and I had this table*, *and this tablet’ and it’s a bouquet*.*”* (Sexual Minority Young Person 13)

Similarly, stakeholders perceived substance use to be part of the culture of LGBT communities and more visible and prevalent. They also acknowledged the positive aspects of substance use.

*“Our culture is somewhat centred around taking drugs and alcohol for fun and for socialising*.*”* (Stakeholder 12)

#### Stereotyping and media bias

Some sexual minority young people were critical about the common perception that LGBT people use more substances than the general population. Sexual minority young people who did not consider there to be any differences between sexual minority and sexual majority populations believed this to be a stereotype about LGBT people and gay men in particular.

*“I don’t think it’s particularly that different […] I guess there is a certain stereotype about gay boys and drugs*.*”* (Sexual Minority Young Person 3)

Similar statements were also made by sexual minority young people who generally agreed that substance use is higher among LGBT people but blamed the media for inflating reports on the levels of substance use among LGBT people, constructing a view that does not adequately represent the communities they experiences.

*“Just*, *I guess I have the perception a lot of people know that it’s there*, *you always see it in the news […] Because of the media coverage and just the general perception*. *So*, *I wouldn’t say it is*.*”* (Sexual Minority Young Person 22)

#### Specific substances

Sexual minority young people also discussed the use of specific substances, making statements about illicit substance use in general or discussing the higher rates of specific substances such as ecstasy [Methylenedioxymethamphetamine], methamphetamine, and poppers [amyl or alkyl nitrite] use within LGBT communities. However, sexual minority young peoples’ perceptions of substance use also appeared to be influenced by media rather than their own experience and observations, particularly for substances commonly used for sexual pleasure (e.g., methamphetamines). These substances were perceived as particularly harmful, even though participants were generally not able to describe how these substances work or how they affect a user’s physical and mental wellbeing.

“Well, from what I’ve heard from a magazine on Facebook […] it’s saying that there’s ICE [methamphetamine] substance abuse at the moment skyrocketing within the community. But apart from that I haven’t really heard much.” (Sexual Minority Young Person 15)

Sexual minority young people also made statements specifically aimed at alcohol use, with most perceiving alcohol use to be more prominent in LGBT communities than the general population. However, these perceptions were mostly based on experiences within night-time economies and licensed venues rather than other activities within LGBT communities.

*“From my experiences*, *there’s a lot of emphasis based around alcohol*. *Especially lesbians in clubs*.*”* (Sexual Minority Young Person 22)

Some sexual minority young people did not perceive there to be significant differences in alcohol consumption and argued that there is a general alcohol-use problem within Australian that is simply mirrored in LGBT communities.

*“I think alcohol is prolific in all subcultures in Australia*. *So*, *I don’t think that’s unique…*.*”* (Sexual Minority Young Person 6)

Some sexual minority young people also addressed tobacco use in their interviews with most perceiving tobacco use to be higher within their peer group and licensed venues.

Community stakeholders also discussed potential difference between substances. They did not perceive a significant decline in substance use within LGBT communities in the recent past, even though such a trend was noted in the general population.

*“More and more people are abstaining when you look at the whole population from drinking alcohol; young people especially than ever before […] I’m not sure if younger people are abstaining as much as their heterosexual counterparts*.*”* (Stakeholder 12)

Furthermore, stakeholders agree that methamphetamine in particular is a significant problem within the community; which was perceived to be epidemic, particularly among gay and bisexual men. Similar to sexual minority young people, stakeholders were most concerned about the use of sexually stimulating substances. However, this might be due to professional work experiences of LGBT health stakeholders.

*“I get concerned about the increasing use of ICE (methamphetamine) amongst young gay people these days*. *And I do know that LGBTI people tend to use a little bit more than their fair share and it can be an issue for some members of our community*.*”* (Stakeholder 5)

#### Higher degree of acceptability

Overall, sexual minority young people perceived a generally higher acceptability of substance use within LGBT communities compared with the general population.

*“I guess for me and the experience that I’ve had it’s been more accepted in the LGBT community*. *It’s not really sort of questioned or anything like that*. *People are like ‘sweet*, *no worries’ and just keep going*.*”* (Sexual Minority Young Person 12)

Sexual minority young people perceived there to be generally high levels of acceptability towards alcohol use in Australian society. However, the acceptability of substance use was not perceived to be equal for all substances, with ‘hard drugs’ perceived to be significantly more accepted within LGBT communities than the general population.

Substance use was generally perceived to be a community norm among stakeholders, which contributed to the higher levels of acceptability of licit and illicit substance use.

*“Drug use is generally quite widely accepted*, *especially illegal drugs […] it depends on the environment but there’s generally more of a culture of tolerance around it*.*”* (Stakeholder 12)

#### Reasons for disparities in substance use

We also asked sexual minority young people to identify reasons for the disparities in rates and acceptability of substance use in sexual minority populations compared to the general population. Explanations and reasons that emerged from the data can be categorised into the following main categories: (a) LGBT culture and life; (b) socio-political and ethical values of LGBT communities, (c) marginalisation, discrimination and mental health, and (d) other reasons. Reasons perceived to be directly related to LGBT communities are discussed first, acknowledging the current scarcity of research in this area and the expert-by-lived-experience status of participant. Reasons identified by sexual minority young people were later presented as a short summary to stakeholders who were asked for their opinion on these reasons based on their past professional experiences in LGBT communities.

### LGBT culture and lifestyle

Sexual minority young people identified a range of factors perceived to be ingrained in the LGBT culture and lifestyle that may contribute to substance use, including the role of substance use in socialising, and peer-pressure within the community.

Role of substance use in socialising. Many sexual minority young people perceived LGBT communities to be largely centralised around licensed venues such as bars, clubs, or LGBT-specific parties, and substance use in general. This effect was perceived to be particularly relevant to the consumption of alcohol even though participants said that the ‘bar culture’ within LGBT communities may also lead to an increase in the use of illicit substances among LGBT people as these are more likely to be accepted in bar and entertainment environments. This assertion was also perceived to be consistent with their own experiences in LGBT venues to an extent they did not experience in mainstream venues.

*“I’d say the culture and the sort of structure of the LGBT community does promote drinking and substance use in some aspect*.*”* (Sexual Minority Young Person 28)

*“There’s someone just like selling it*. *Just running around and selling it*.*”* (Sexual Minority Young Person 13)

As a result, sexual minority young people expressed a strong desire for more social options within LGBT communities to meet other LGBT people without a focus on alcohol use or a perceived constant pressure to consume alcoholic beverages. Such an option was perceived as particularly important for underage young people due to their inability to enter licensed venues. The atmosphere in licenced venues and alcohol-related night entertainment was also generally doubted to be ‘appropriate’ for young people.

*“We would often have difficulties finding activities that everyone would be able to participate in quite comfortably*, *when people wanted to drink and some didn’t want to drink because it was illegal*, *they were 17*, *or they just didn’t feel like it was their cup of tea*.*”* (Sexual Minority Young Person 27).

Peer pressure. As a result of the participants’ perception of the current physical structure of LGBT communities to be concentrated around licenced venues and night time entertainment, many sexual minority young people felt that the community generally had a strong focus on celebrating and partying, which ingrained substance use in the community culture itself.

*“‘Cause some part of the LGBT community*, *like there’s a lot of ‘here we are*, *we’re gonna celebrate’ and there is in clubbing scenes*, *there’s a lot of substances*.*”* (Sexual Minority Young Person 5)

Substance use was also seen as a necessity, an activity that facilitates access to and fosters a sense of belonging to LGBT communities. It was also argued that alcohol and other substances are a common occurrence in these environments.

*“It’s like a discourse*, *it’s like your access card to the community*.*”* (Sexual Minority Young Person 2)

In this context, peer pressure to drink and use other substances was considered to be considerably higher in LGBT communities by the majority of sexual minority young people in comparison to the general society. However, peer pressure was perceived to be a passive rather than an active behaviour exercised in an atmosphere in which substance use is common.

*“Like*, *if there’s anyone not drinking*, *it’s kinda weird*. *Like you’d never push it or anything but you are like ‘Uh*, *not drinking*, *what are they’ and they usually get bored and go home*.*”* (Sexual Minority Young Person 3)

Overall, community stakeholders agreed that the higher concentration of licensed venues in LGBT communities may lead to higher levels of substance use among sexual minority people. In addition, participants believed that most community activities are related to alcohol or the nightlife industry which may be harmful for younger people in general. It was also seen as potentially exacerbating the integration and participation of LGBT young people and minors into LGBT communities by restricting their access to many LGBT community venues and events. Other community events related to sex-on-premises venues were not perceived to be an alternative for young people due to their strong focus on sexual activities. Stakeholders were reminded of their own introduction into LGBT communities by these statements. They perceived licensed venues to be one of the very few options for LGBT people to socialise with peers.

*“I do agree with the younger ones*. *If they’re under 18 there’s really nowhere to go besides the one organisation […] And I’ve been that person as well*, *I was literally not a big drinker […] suddenly I would drink at a nightclub*, *because that was where I went*, *that was the only place I could go and that’s what it is*.*”* (Stakeholder 1)

### Socio-political and ethical values of LGBT communities

During the interviews, it was apparent that sexual minority young people also perceived there to be differences in the ethical and socio-political values, cultural norms and religion, and liberty of LGBT people and their non-LGBT counterparts. These differences were not seen as a result of the perceived higher concentration of alcohol-related venues and activities in LGBT communities but rather as a result of the unique experiences of LGBT people such as rejection, marginalisation or coming out.

Cultural norms and religion. Sexual minority young people generally perceived LGBT people to be less bound by the religious norms and cultural standards of the general community which result in substance use being seen as a deviant behaviour by the general population. The lower connectedness of LGBT communities to these norms and standards may explain the higher acceptability and extent of substance use.

*“It generally depends on the culture thing too*, *because most of the GLBT people don’t conform to culture standards and they are not really religious and there’s not much holding them back I guess*, *so there’s definitely a slightly higher use*.*”* (Sexual Minority Young Person 1)

Sexual minority young people perceived the cultural differences between LGBT communities and the general society to be positive and liberating but did not consider this to be used as an excuse for engaging in illicit substance use. However, some did not perceive these differences in substance use norms negatively.

*“But we’re used to being different from the norm*, *so why can’t we do the same thing with alcohol*?*”* (Sexual Minority Young Person 27)

Liberty. Besides not adhering to cultural norms, sexual minority young people also perceived LGBT communities to have a different set of socio-political and ethical values with a stronger focus on tolerance and liberty than the general population. This was assumed to be a result of on their own experiences of belonging to a marginalised minority.

*“My opinion is that because we’ve experiences other people telling us what to do with our lives*, *a lot of LGBT people are very hesitant to say to someone ‘Well*, *you shouldn’t do that*.*’”* (Sexual Minority Young Person 31)

These personal experiences lead to a decidedly more liberal community than the general population. This environment was perceived to be more likely to accept alternative lifestyles and tolerate the use of illicit and harmful substances. Some sexual minority young people perceived the environment to be free of judgement in terms of substance use, in which questioning a community members’ substance use behaviour could be seen as disrespectful or even discriminatory. Sexual minority young people also felt that this acceptance goes beyond substance use behaviours, and that LGBT communities are generally more accepting towards behaviours deemed illegal or simply not part of the mainstream culture.

*“Anything that’s probably not mainstream or not legal because of their acceptance of difference amongst the LGBTI community that I think drug use is also accepted more so than the general populace*.*”* (Sexual Minority Young Person 6)

Stakeholders were less conclusive regarding the impact of perceived socio-political and ethical values of LGBT communities. Some stakeholders agreed with the perceptions of sexual minority young people and thought the overall liberal socio-political and ethical values of LGBT communities contributed to the greater burden of substance use in the communities. The discourse on substance use behaviour within LGBT communities was assumed to differ significantly from that of the general population, potentially leading to a different socialisation of community members.

*“Yes*, *yes*, *definitely yes*. *We don’t tell people how […] to live their lives because we do get that so much from the outside world*.*”* (Stakeholder 13)

While other stakeholders agreed that LGBT communities are generally more liberal, they did not consider this to be a significant contributor to the substance use disparities. Rather, they argued that the communities ignore the problem and refuse to have a discourse about problematic substance use behaviours. Some thought young people may confuse this limited discourse with liberty; even though this is in contrast with the notion of LGBT communities being open minded about differences and other behaviour.

*“I agree with the fact that we are very inclusive with our political views […]*. *But when it comes to lifestyle choices […] like that stuff is usually kept either as gossip or kept quiet […] we kind of just accept that’s how it is and we don’t actually reach out to people*.*”* (Stakeholder 14)

One stakeholder suggested that this liberty might not be politically motivated but rather a result of limited knowledge regarding the potential effects of substance use on the communities. However, this view was not expressed by other participants who argued that knowledge about the effects of substance use is high within these communities.

*“I think also it’s not so much the attitude*, *I think that the knowledge in the community about the effects*, *about the harm of substance use*. *I think that’s not really too big*.*”* (Stakeholder 10)

### Marginalisation, discrimination & mental health

Nearly all sexual minority young people in this study identified marginalisation and discrimination as major factors contributing to the substance use disparities in LGBT communities. Four subcategories emerged from the data for this category: being different, isolation and belonging, discrimination, and mental health.

Being different. Sexual minority young people perceived marginalisation based on their sexual minority identity as a key contributor to identity issues and struggles among sexual minority young people. Identifying with a different sexual minority identity was seen as a major stressor in the everyday lives of sexual minority youth. Substance use was perceived to be a maladaptive strategy for coping with being different to the majority of the population.

*“When I identify different from the majority it does make life much harder*. *So*, *I don’t consider it a rational choice*.*”* (Sexual Minority Young Person 26)

However, not all sexual minority young people perceived being different to be a problem in Australia. One sexual minority young person perceived acceptance not to be a current issue and did not think it had a negative influence on substance use within today’s LGBT communities.

*“In the past I would have said it would have been because they weren’t allowed to be themselves*. *But in my generation and people even younger than me […] would have no issue being accepted in Australian culture at all […]*. *They wouldn’t be using substances to hide up who they are”* (Sexual Minority Young Person 20)

Isolation and Belonging. Having a minority sexual identity may lead to isolation and conflict with friends and families. Several sexual minority young people described the difficulties of coming out to their friends and family members and fears these significant others having negative perceptions of their sexual identity. Substances use is viewed as an emotional escape from these situations and feelings.

*“From my experience a lot of the LGBT people have more worries about their sexuality*, *about how they are perceived and seen by their peers or family or friends*. *These worries seem to kind of slip away if they are drunk*, *they are high or they are cooked up*.*”* (Sexual Minority Young Person 4)

Discrimination. Discrimination and marginalisation based on their sexual orientation were identified as major contributors to substance use. Sexual minority young people described experiencing challenges with homonegativity and bullying throughout their lives, but finding this particularly difficult during adolescence. Micro-aggressions such as the usage of heterosexist language (‘that’s gay’) or the endorsement of heteronormative culture by people with a sexual majority identity in their everyday lives were experienced as a form of discrimination. Discrimination was also identified as a contributor to mental health issues and the use of substances to cope with these.

*“I feel like it’s a bit stressed*. *[…] I think LGBT people are already experiencing more stress than straight people struggling with […] other people’s reactions*, *something like bullying*, *name calling*, *things like that*. *I think it definitely gives a different reason for them to use substances*.*”* (Sexual Minority Young Person 26)

One sexual minority young person perceived the lack of programs targeting substance use in LGBT communities as a form of discrimination.

*“It seems to be less acceptable now outside because they’ve got all those programs targeting*, *you know*, *young men that go out and drink a lot*. *Whereas they don’t have that for us*. *Inside the community*. *In a way*. *You know*, *it’s not mainstream*. *So I think it is just seen as more acceptable*.*”* (Sexual Minority Young Person 21)

Mental Health. Sexual minority young people linked their experiences with being different, their feelings of isolation and lack of belongingness, discrimination and marginalisation with mental health problems among people with a minority sexual identity. They also perceived the prevalence of mental health issues to be higher within LGBT communities.

*“There a higher prevalence of anxiety*, *depressive disorders*, *and that kinds of things just because of what we have to go through […]*. *People definitely don’t get that outside of the community*.*”* (Sexual Minority Young Person 22)

Sexual minority young people assigned two roles to substance use: coping mechanism as well as self-medication. Self-medication was seen as a viable alternative to seeking help or a prescription from a medical professional to avoid exposure to further stigmatisation.

*“There is a higher instance of them use the illegal substances to self-medicate because they are already dealing with too much*. *They [medical professionals] tend to lump it all under the same thing ‘I am sad because I am gay’ […] sort of thing*. *It got nothing to do with that*. *It’s underlying*.*”* (Sexual Minority Young Person 18)

Stakeholders generally agreed that marginalisation, discrimination, and mental health issues are important drivers for substance use, which represents a coping mechanism for coming out problems, internalised homonegativity, stigma, and resulting mental health problems which are perceived to be more prevalent in LGBT communities.

*“It’s an incredibly important factor*, *because I feel people only take drugs and alcohol to a dangerous point*, *so excessively*, *when they’re not coping with stresses in their life*. *And generally*, *people who are gay*, *lesbian and transgender have a lot of struggles*.*”* (Stakeholder 10)

### Other reasons

During the interviews, sexual minority young people mentioned a range of other factors that could not be assigned to one of the three preceding main categories. These were only mentioned by a small number of sexual minority young people.

Media influence. Participants also criticised LGBT media for their portrayal of LGBT communities as consistently celebrating as well as regularly using and abusing substances. This image might normalise substance use in LGBT communities, and can have a negative impact especially LGBT youth.

*“I think media has played like a big part in the acceptability of substances because […] like there’s not a lot of gay and lesbian movies out there already but the ones that are out there*, *and shows like Queer as Folk and stuff*, *like they just really show casual drug use especially among gay men […] like it’s no big deal*. *So*, *I do feel like it’s just one of those things that people are like ‘Oh*, *yeah*, *it’s just part of the party culture*, *the gay party culture or whatnot*.*”* (Sexual Minority Young Person 31)

Inherited behaviour. It was argued that most problems in LGBT communities were greater in the past. While sexual minority young people perceive current levels of substance use within LGBT communities to be lower than a few decades ago, substance use behaviours are perceived to have survived these changes to some extent.

*“But for LGBT community structure I think that trickling down from difficulties older generations had where they’d been stigmatised”* (Sexual Minority Young Person 28)

Demographic differences. LGBT communities were perceived as being younger than the general population on average as a result of fewer older people coming out as LGBT. Respondents saw this as a reason for the greater acceptance of substance use in LGBT communities, since young people are generally more accepting towards substance use.

*“I think substances are more accepted in the younger generation*. *Things like that*, *it’s sort of more accepted*.*”* (Sexual Minority Young Person 10)

Furthermore, it was suggested that LGBT people have more disposable income and were less likely to have children, allowing them to spend a higher portion of their income on visiting licenced venues and substances, and to worry to a lesser extent about the consequences substance use can have on their family.

*“I would just say in my experience it’s more acceptable within the gay community to have more illegal substances because it is not as much responsibility compared to my straight friends […] I would say it’s not as high as in the gay (community) and I put that down responsibilities […] I would say children and partnerships for sure*.*”* (Sexual Minority Young Person 8)

Conflicting identities. Two sexual minority young people perceived that certain identities such as religions or cultures may be perceived as being in conflict with a person’s sexual identity. These perceived conflicts may result in higher levels of stress in which substances may be used as a coping mechanism.

*“Using substances to escape from that feeling*, *trapped between the two different sides of your identity*, *using substances to try and forget about one*. *One side of your personality is telling you that the other side is a really terrible thing*.*”* (Sexual Minority Young Person 30)

### Potential interventions

Stakeholders were also asked to identify potential interventions for reducing substance-related harm in LGBT communities. A range of potential interventions and demands were identified targeting politics and legislation, NGOs and existing activities/groups, commercial LGBT venues, as well as LGBT communities themselves.

### Politics and legislation

Heteronormativity and discrimination were identified as the main contributors to the elevated levels of substance use issues in LGBT communities. Stakeholders highlighted the need for more specific counselling services where LGBT young people could feel safe addressing these issues, as well as more safe spaces for LGBT young people to interact in the community. More funding would be required to increase the capacity of existing services and develop new programs to achieve this. Public funding would also be required for LGBT communities to hold alcohol-free events and activities.

*“I guess it all depends on what sort of funding that the NGOs have for a start*, *we’d love to open up our space to have a coffee shop or something but without money you can’t really do that*.*”* (Stakeholder 1)

Current laws were perceived to marginalise LGBT people and were considered to be key contributors to substance use issues. Stakeholders argued that amending these laws to further normalise sexual minority identities in the general population, would have a significant positive effect on the health of sexual minority youth including reductions in substance use. Three main legal issues were identified: marriage equality, the unequal age of sexual consent, and the so-called ‘gay panic defence’ for violent crimes in the state of Queensland, Australia.

Marriage equality was named most often by stakeholders, as a way of promoting higher levels of acceptance and in return lower levels of isolation and marginalisation.

*“I think politicians need to step up and go with the marriage equality thing because then I think that trickles down to the community that they’re an accepted part of society […] but I think for young LGBT people or even older LGBT people it will be a start in that area where their mental health may improve because they actually feel like that they’re now part of the society they live in*.*”* (Stakeholder 1)

However, while stakeholders generally perceived a change in this area as beneficial for LGBT communities, some also identified concerns about how the current focus on marriage equality could contribute to more problems in some LGBT communities.

*“I think marriage equality is great; let’s do it*. *But I think as a result*, *look at the damage that we are doing to the community as well […] we’re setting ourselves to be*, *if you’re not white*, *in a relationship*, *thinking about children*, *wanting to get married and have a double income with a mortgage*, *all that sort of stuff*, *being the classic ‘successful gay’*, *then you’ve somehow failed […] but the bottom line is*, *a lot of us aren’t that ‘successful gay’ image and that in itself can cause stress*, *anxiety and mental health problems which can then impact on people’s drug and alcohol use; so*, *it’s a vicious cycle*.*”* (Stakeholder 12)

Furthermore, the state of Queensland, Australia’s unequal age of consent was perceived as criminalising gay behaviour hindering the normalisation of gay and bisexual sexuality and identities. The legal age for most sexual activities in Queensland is 16 whereas the age of consent for anal intercourse is 18 years [41].

*“In Queensland we’ve got the problem that we’ve still got a disproportionate age of consent when it comes to anal intercourse*, *as long as there is someone who is willing to slag off in public or in legislation what someone thinks is normal for them*, *they are going to feel marginalised and discriminated against and they are going seek ways accepting themselves*. *And unfortunately*, *I think that means drugs and alcohol*.*”* (Stakeholder 7)

Finally, the so-called ‘gay panic defence law’ was raised as a concern. This legal defence, a part of Queensland’s criminal Code [41], allows a defendant accused of murder to raise the defence that they were provoked by an unwanted homosexual advance which will, if raised successfully, reduce a murder charge to a manslaughter charge. This defence was used successfully as recent as 2008 [42].

*“I think there is a lot more to go*, *you know*? *Changing the laws in Queensland*, *there is the gay panic defence law […] that would make a huge difference in terms of government seeing sexuality and gender stuff is okay for young people*.*”* (Stakeholder 10)

Besides these LGBT-specific legislations, stakeholders also commented on more general legislation related to substance use. Stakeholders perceived current laws criminalising substance use as outdated and a risk to harm minimisation processes.

*“Anything that prohibits discussion*, *that criminalises and stigmatises drug and alcohol use actually makes it worse for those people that are having trouble and are struggling to seek support*. *It goes underground and people don’t seek help and support around that stuff and don’t practice risk-minimisation as much because the shame increases*.*”* (Stakeholder 10)

Similarly, stakeholders criticised the current licensing laws in Queensland which prohibit serving alcoholic drinks at specific times and require licensed venues to close at certain times. This legislation was perceived as being counterproductive and unrealistic. Furthermore, it was assumed that these laws may be able to destroy parts of LGBT communities due to its concentration of licensed venues, effectively destroying safe spaces for sexual minority people.

*“They’ve regulated it and regulated and regulated but there is still no way to stop me*. *Let’s say I’m a conflicted alcoholic and I’m in a situation of possible domestic violence […] you can draw your hours back to 6PM if you want*, *there is nothing that’s going to stop me from buying whatever it is that is my poison in a bottle and bring it home […] and bashing the shit out of my partner […] You’re cutting the hours in which they can associate with their own people and forcing them into a private environment where they are either going to escape alone or with somebody who they may be able to handle when they hit a point too far*.*”* (Stakeholder 3)

### LGBT NGOs and existing activities & groups

The majority of stakeholders were able to name non-alcohol related activities and groups in LGBT communities but considered these activities and groups to be difficult to find. More advertising was needed to make sure people, particularly young LGBT people, know these activities and groups exist.

*“Well*, *I guess you need to advertise where people are going to*, *maybe things like Grindr or Pink Sofa for girls or I guess there’s the LGBTI media as well*.*”* (Stakeholder 5)

A further integration of social media to connect LGBT people and raise awareness around projects and activities in the communities were seen as particularly important. Social media in general was perceived to be a greater responsibility of young community members because social media was recognised to be more natural to and more used by this demographic.

*“Social media*. *I think that’s a very fair call*. *I mean they’ve grown up in a world that is totally different and I’m sure technology will advance beyond where it is now*.*”* (Stakeholder 3)

Even though some activities already exist, stakeholders felt there was a need for more social activities in the communities rather than just in licensed venues. Such events would allow people in the communities to socialise meaningfully and to strengthen community connectedness.

*“We have to try to come up with new ways to get people to connect that’s centred around other things […] so maybe organising*, *getting more social activities like that where there is a common focus that doesn’t require any alcohol or any substance*.*”* (Stakeholder 6)

Social media was also seen as a potential way to educate and engage LGBT people, particularly young people about substance use, health and the culture of LGBT communities.

*“Social media*, *especially with YouTube*, *Facebook*, *Tumblr which basically engage more and more young people […] and getting their information from social media*. *[…] Social media is more and more accessible to community groups and service providers and educational messages*. *A really good effort to push our story-telling and sharing and information in a positive way through those mediums would actually have a huge impact on creating awareness for young people around drug and alcohol use*.*”* (Stakeholder 10)

The current focus of NGOs in the communities were considered to be too narrow and stakeholders felt they could take better care of LGBT young people in the communities in the way mainstream organisations in the general population do.

*“I think we just need a lot more places for the kids to go to and to support the existing services that are there […] The reason why you have big football clubs is because they look after their community*, *you know*, *representation*. *And they keep behind them right up to senior league*. *And if we only concentrate on one area […] we can’t wait for them to get to this*, *because they’re fucked when they get there*.*”* (Stakeholder 9)

However, stakeholders also perceived a lack of professional services related to alcohol and substance use customised to the needs of the communities. Similar services existed in the past but are still needed.

*“I guess providing services as well*, *like you know*, *we had the ATODS [Alcohol*, *Tobacco*, *and Other Drugs] program for our community and I think that was a really important program*. *I guess*, *not to preach to people about what to do but if they’re looking for support or help it’s there and available for them*.*”* (Stakeholder 5)

Similarly, mainstream services were criticised as being insufficiently trained and educated to take care of members of LGBT communities. Community NGOs were considered to be responsible for training and educating other services and health professionals to help them better understand the unique health issues of LGBT communities.

*“If it’s going to be a mainstream program that’s running these types of services*, *they need to be inclusive*, *they need to show gay people in their campaigns*, *they need to target gay venues*, *community groups and media about their program and I just don’t see that happening really*. *I guess if they’re not going to fund gay community groups to provide it*, *they need to consult and learn with gay community groups to be more inclusive*.*”* (Stakeholder 8)

### Commercial LGBT venues

Commercial LGBT venues were considered to have a limited potential role in addressing substance use in LGBT communities. LGBT venues were seen as profit-driven and generally not interested in either alcohol-free events, due to their lower profit margins, or LGBT young people, due to their lower levels of disposable income

*“Most of my work with these venues*, *they’ve always been incredibly profit-driven*. *Community yes*, *but they don’t want to interfere with anything that interferes with their profit margins*. *[…] There’s not really much of an incentive for them financially to provide services or events that are non-alcohol for younger gay*, *lesbian*, *and transgender people*.*”* (Stakeholder 10)

However, commercial venues were identified as valuable environments for advertising existing services and raising awareness of the issue at hand.

*“I believe you need to have more advertising […] like they did with smoking*. *[…] And I think in the gay community there needs to be some sort of*, *how do I put it*? *I mean advertisement but how do they get it to the people as well*, *they can only do it by advertising at the clubs*, *like the pubs or sex venues*.*”* (Stakeholder 11)

Stakeholders also recognised the potential benefits of collaborations between NGOs and commercial venues for developing and promoting safer experiences in the nightlife economy.

*“I tend to agree that peer pressure would lead to a lot of use of drugs and alcohol*. *If there’s a community [*..*] that don’t want to go to venues and don’t want to partake in that sort of drugs and alcohol side of things*, *they need to find a way […] if they can go to an LGBT venue and say ‘Look*, *we want to have something here that’s not alcohol or drug related*.*’ and see if that venue will get behind them […] I think there’s got to be a bit of collaboration between venues and those in the community*.*”* (Stakeholder 1)

### LGBT communities

Stakeholders identified a number of ways to address substance use in LGBT communities by changing the culture within the community. LGBT communities were often seen as having lost a part of its culture and solidarity in the past.

*“I think that the gay and lesbian community needs to do something about it and I don’t think that they’re willing to do anything about it*. *I think our community has lost its heart 100%*. *I’ve seen a steady decline in our community […] but our whole community can’t be assed about doing anything at the moment*. *I feel sorry for our community; I think we’ve lost our way*. *Sadly*.*”* (Stakeholder 2)

A need to change and renew the culture of LGBT communities was identified as a way to increase the sense of community as well as community cohesion. The need for discourse on community values, and for an open and genuine conversation about the problems and issues facing LGBT communities and how to solve them, were highlighted as one way of increasing community solidarity.

*“And all this [substance use] has all become quite a big issue and the epidemic has happened because of a lack of conversation*. *[…] A topic that keeps coming up over and over and over again but there’s no solution because it’s not handled the correct way*. *I think a conversation needs to happen openly in a public forum without biases amongst people involved*. *That would really help*.*”* (Stakeholder 14)

Furthermore, the isolation of some LGBT communities from the general population and the activities and services available within it were identified as impediments to help seeking in sexual minority people

*“We are so head-down gay*, *gay*, *gay*, *gay*, *gay*, *gay*, *gay*, *gay*, *gay; it doesn’t really exist*, *outside of the gay culture*. *We actually close ourselves off to anything beyond that gay culture of going to a nightclub and drinking; there is nothing else*.*”* (Stakeholder 14)

The perceived loss of community or perceived reduction of LGBT communities to a nightlife economy was linked to the overall improvement of LGBT people’s health and increased socio-political opportunities. Stakeholders saw a lack of purpose, particularly for young people, as responsible for a decline in the communities.

*“I think they’re*, *the younger community is a community that hasn’t got a purpose*. *We all had a purpose*, *in the 1980s and 1990s we were all fighting for the cause*. *It was HIV that we were all fighting against*, *and discrimination*, *and about keeping and surviving of our community*. *And in the noughties [sic] it was a little bit better and we learnt how to party a bit more*. *And then we lost our mojo*.*”* (Stakeholder 2)

This perceived loss of community was discussed by most stakeholders; however, not all stakeholders agreed that the community is generally being lost, arguing that the current disruptions are simply a result of a larger transformation and restructure of LGBT communities by including mobile dating applications, and other internet-based services and communities as well as the potential of LGBT groups to meet in venues not necessarily identified as an LGBT venue due to a heightened feeling of safety in public spaces.

## Discussion

Participants perceived the use of licit and illicit substances to be a significant and meaningful problem within LGBT populations compared to the general population. Sexual minority young people were particularly concerned with the levels of substance use within their communities. However, it appears that young participants at times identified any substance use–particularly of illicit substances–to be problematic and harmful, whereas community stakeholders had a broader perspective on the issue. These differences in perceptions might be explained by recent research showing that young people in Australia are less likely to engage in, accept or approve of substance use [4, 43–45].

Sexual minority young people, as well as stakeholders, saw marginalisation, discrimination and mental health issues as key contributors to the disparities in substance use between sexual minorities and their heterosexual peers. Substance use among sexual minority young people was perceived to be a strategy for coping with these issues. These findings are consistent with the current literature showing that LGBT people are exposed to higher levels of physical and verbal violence [46], as well as other forms of discrimination, marginalisation and oppression in an environment generally perceived as heteronormative [12]. Furthermore, these findings are in line with substance use often being used as an avoidance-based strategy to address psychological distress [47]. They are also consistent with LGBT specific minority stress models negative health behaviours among sexual minority groups are a mechanism to cope with living in a heterosexist and homonegative environment [12].

Participants were indifferent about the influence of socio-political and ethical values of LGBT communities on substance use. Sexual minority young people generally argued that community values are strongly correlated with both the higher acceptance and use of substances in the communities. LGBT communities were also perceived to foster more liberal and open-minded lifestyles, including substance use, than other communities [48]. Previous studies have shown that people with more liberal political beliefs are more accepting of substance use [49] and are more likely to engage in substance use [50]. However, stakeholders had more mixed views about the potential impact of the liberal political values of LGBT communities on substance use. While they perceived LGBT communities to be very inclusive, open-minded, and liberal, the majority did not agree that this liberty also extended to substance use behaviours. The liberal attitude towards substances perceived by sexual minority young people might be related to other potential factors such as the general lack of discourse on substance use behaviours within LGBT communities, as well as low levels of education regarding the possible consequences of substance use. Recent research among gay and bisexual men in Australia has also shown that attitudes towards illicit substance use are more heterogeneous than expected, showing methamphetamine or heroin use are less acceptable than cannabis or ecstasy use [45].

Consistent with previous research, LGBT communities were also seen to be less bound to cultural standards and religious norms than other communities or general society [50–53]. Previous studies have shown the point at which substance use is considered a deviant behaviour among the key sources of socialisation in other minority communities exerts a strong influence on an individual members’ perceptions of substance use at which point substance use can be constituted a deviant behaviour [54, 55]. Available literature argues that LGBT communities are main sources of socialisation for LGBT people [17, 56] and the more liberal attitudes toward substance use may explain the higher levels of acceptability and rates of substance use within these communities. The use of substances, particularly alcohol, may be linked with expectancies to increase the potential to be further included in LGBT communities [57]. However, past research with gay and bisexual men shows that both very high and very low levels of identification and participation within LGBT communities can be associated with elevated levels of substance use [21, 22]. These results suggests that socialising within LGBT communities does not automatically lead to higher levels of substance use, and that a range of other factors are likely to impact on this relationship. Participants also criticised the fixation of their communities on licensed venues as a key contributor to the elevated levels of substance use, as it increases access to and peer-pressure to use substances. The current literature supports these findings that the concentration of licensed and entertainment venues in the communities, and the constant availability of alcohol may increase substance use [24, 27].

Other reasons for substance use within these populations commonly discussed in the current body of literature such as sexual sensation seeking among gay and bisexual men [6, 7] were mentioned by very few participants and were not perceived to be a significant factor. This might be a result of the diversity of the sample including only 13 participants identifying as male as well as the overall low age of participants in the sample.

Several potential interventions to reduce the burden of substance use on these communities were proposed by the stakeholders. Existing laws were identified as discriminating against LGBT communities. These included the lack of marriage equality in Australia as well as the unequal age of consent and the gay panic defence in Queensland at the time of data collection. These laws were perceived to interfere with the normalisation of sexual minority identities and to further marginalise LGBT people. However, the Queensland Parliament recently equalised the age of consent in the meantime [58] and abolished the gay panic defence laws [59]; Australia has furthermore legislated marriage equality in the meantime [60]. Literature shows that legal equality and advanced civil rights can have a positive influence on the mental health of LGBT people [61] which potentially could reduce the burden of substance use within this population. Similarly, participants argued that current legislation regarding alcohol and substance use is counterproductive. Some participants thought the legalisation or decriminalisation of substance use would help foster an open discussion about substance use in LGBT communities. A few countries such as Portugal have made meaningful advances reducing the burden of substance use with interventions including elements of legalising or decriminalising substance use [62]. Existing licensing laws were also identified as unhelpful and potentially reducing the already small number of safe places for the LGBT young people in the community. However, the State Government of Queensland foreshadowed new liquor licensing laws at the time of the interviews, including earlier last drinks and a ban of ‘rapid intoxication drinks’ [63]. The importance of these laws might have been inflated due to extensive public discussion at the time.

Beyond changes in legislation, stakeholders also stressed the need for more funding to provide services and activities targeting substance use in the community. Services and activities related to substance use in this population were perceived to be best delivered by LGBT community NGOs with expertise in the area. These NGOs were also seen as potential providers of non-alcohol related activities and events for young LGBT people. While mainstream organisations offering substance use services were criticised for their lack of expertise with the specific circumstances of LGBT people [64], the responsibility of providing training and education for such services was seen to reside with community-based LGBT organisations.

Advertising through social media was seen as a key strategy for increasing community awareness of the prevalence of substance use and the existing services available to address it. Previous research has stressed the potential of social media for marginalised communities [65]. The role of commercial venues in curbing substance use problems in LGBT communities was perceived to be limited to collaborations with community groups and organisations as well as advertising for existing services and activities. However, the need for a greater discourse on substance use within the communities was identified as an important way of developing a community-wide response to problematic substance use.

### Strengths and limitations

Several steps have been undertaken to strengthen the reliability of this analysis, including an LGBT-specific ethical strategy in planning and conducting interviews, and the use of strict verbatim transcriptions as well as internal and external reliability checks. Analysing interviews with two groups, sexual minority young people and self-identified stakeholders of LGBT communities, is a further key strength of this analysis, providing a multifaceted perspective on substance use within this population. In addition, this approach strengthens the validity of results by combining experiences and perceptions of young people with the perspectives of a highly experienced group of community members. It furthermore goes beyond the descriptive nature of the currently available body of literature on substance use within this hard-to-reach at-risk population.

However, this study has some limitations. Sexual minority young people in this study had comparably high levels of education, 61% of these participants identified as gay or lesbian, mostly identified as White or Caucasian and only two of the 14 stakeholders identified as female. Furthermore, no person under the age of 18 years responded to advertisements. This limitation indicates not all LGBT communities were adequately represented in the current study which may have excluded some of its most vulnerable members. The semi-structured nature of the interviews might have also influenced participants’ responses. It is also unclear to what extent participants’ own experiences with substance use may have influenced their perceptions of the acceptability of substance use within LGBT communities even though no clear pattern was detected in the analysis. The analysis also showed that participants had different levels of LGBT community experience, and those with low levels of experience may not be able to adequately or realistically describe the substance use culture within these communities. Similarly, participants with very low affiliations to the general community may not be able to adequately compare substance use within sexual minority and sexual majority young people.

## Conclusion and implications

The findings of this study suggest that sexual minority young people are aware of the high levels of acceptability and elevated rates of substance use within their community. Some sexual minority youth perceived the substance use culture in LGBT communities to be such a significant problem they had estranged themselves from it. A range of factors which may contribute to these disparities in substance use were identified including marginalisation, discrimination, mental health as well as the socio-political values of LGBT communities, the physical structure of LGBT communities and demographic differences between LGBT communities and the general population. Stakeholders overall agreed with the viewpoint of sexual minority youth; however, they were not as conclusive regarding the effect of the LGBT communities’ liberty on substance use assuming that these might be related to a lack of interventions and substance use specific knowledge in communities, misinterpreted as liberty. Stakeholders further identified a number of potential interventions to reduce the burden of substance use on this population targeting legislation, existing services and activities, commercial LGBT community venues, as well as the overall culture within LGBT communities.

### Implications for future research

Previous research has tended to focus on the role of mental health, discrimination, and minority stress to explain disparities in substance use. This research highlights the importance of targeting a wider range of factors within LGBT communities to reduce substance use. Future research should investigate the influence of the socio-political and ethical values towards substance use in LGBT communities, as well as the role of substance use and the community itself in the socialisation process of LGBT young people. The results stress the importance of recognising a range of perspectives in research on LGBT community specific topics, including community stakeholders, considering their experience and involvement within the communities.

### Implications for practice

This research highlights the importance of tailoring substance misuse interventions to the culture, socio-political and ethical values of LGBT communities. A need for safe spaces and activities in LGBT communities not reliant on substance use to facilitate socialisation was identified as well as a more open discourse on the impact of the substance use culture within LGBT communities on substance use behaviour among LGBT youth. Addressing the representation of substance use in media and marketing campaigns targeting LGBT communities could have a lasting positive effect on the community and the health of its young people. The importance of the community for LGBT people, particularly LGBT young people, highlights the need to increase the availability of more substance use treatment services. The provision of specialised training to mainstream services is needed to raise awareness of the specific needs of LGBT people and might help to reduce current barriers to accessing them. Furthermore, LGBT communities should endeavour to provide more safe spaces without a focus on alcohol and other drugs, particularly for young LGBT people.

## Supporting information

S1 File Consolidated criteria for reporting qualitative studies (COREQ): 32-item checklist. (DOCX)
